# Chile's agricultural research institute plant tissue analysis dataset

**DOI:** 10.1016/j.dib.2023.109442

**Published:** 2023-07-23

**Authors:** Fabio Corradini, Francisco Casado, Verónica Rojas

**Affiliations:** Instituto de Investigaciones Agropecuarias, INIA-La Platina, Santa Rosa 11610, Santiago 8831314, Chile

**Keywords:** Plant analysis, Plant nutrition, Nutrient concentration, Agricultural research, Chilean endemic species

## Abstract

This dataset holds 9,175 entries that report the nitrogen, phosphorus, potassium, calcium, magnesium, zinc, manganese, copper and boron contents of various plant species, with a focus on crops. The dataset accounts data of 94 plant species, and present nutrient concentration of 14 different plant tissues. The data are derived from the Soil and Plant Nutrition Lab of the Chilean Agricultural Research Institute, which provided services to farmers in the Chilean Central Valley between 2006 and 2020. The analytical methods used to generate these data were consistent across all years, ensuring the reliability of the information. Specifically, nitrogen content was determined using the Kjeldahl method, while all other analytes were quantified via colorimetry (phosphorus and boron) or atomic absorption spectrometry following high-temperature oxidation and dilution of the ashes with hydrochloric acid. The dataset has numerous potential applications, including the estimation of crop nutrient extraction rates, the identification of nutrient deficiencies or excesses, and the provision of reference or prior information for researchers studying plant physiology. The dataset includes 21 Chilean endemic species, which might be of particular interest to researchers studying the biodiversity and ecology of Chile's Central Valley.

**Specifications Table**

**Table:** 

Subject	Agronomy and Crop Science
Specific subject area	Crop nutrition
Type of data	Table
How the data were acquired	The dataset comprises plant tissue analysis for total nitrogen (%), phosphorus (%), potassium (%), calcium (%), magnesium (%), zinc (mg/kg), manganese (mg/kg), copper (mg/kg) and boron (mg/kg). Total nitrogen was measured by Kjeldahl method (Gerhardt Kjeldatherm, Turbosog and Vapodest 50 s). The remaining elements were measured after high-temperature oxidation of the organic matter and dissolution of the ash with hydrochloric acid. Digest analyte concentrations were determined by atomic absorption spectrometry (Thermo Scientific iCE 3000). Phosphorus and boron were determined by spectrophotometric methods: vanadium phosphomolybdate and azomethine-H methods, respectively (Perkin Elmer Lambda 3B).
Data format	Raw
Description of data collection	The data were pulled out from laboratory reports issued between 2006 and 2020. Reports were gathered from the laboratory archive. Reports were included in the dataset whenever they: (a) had plant tissue data measured with the methods indicated above; (b) included samples from Chile's central valley.
Data source location	• Institution: Instituto de Investigaciones Agropecuarias, INIA • City/Town/Region: Santiago, Región Metropolitana • Country: Chile • Raw data sources: Laboratory reports of La Platina Soil and Plant Nutrition Laboratory issued to customers between 2006 and 2020 accounting for samples collected in Chile's central valley (32°55 – 35°01 Lat; 70°01 – 72°25 Long)
Data accessibility	Repository name: Biblioteca Digital INIA Data identification number: 68,967 [1] Direct URL to data: https://hdl.handle.net/20.500.14001/68967

## Value of the Data

• The dataset can be used to estimate crop nutrient extraction and set out fertilization plans, benefiting farmers, agronomist, and scientists.
• The dataset can be used to estimate deficiencies or excesses ranges for different crops or set out likelihoods in future analyses or studies.
• The data set can be used as a reference by researchers studying plant physiology and nutrient metabolism.
• The dataset can serve to set out priors in Bayesian calculations with new data.
• Plant tissue analysis data on crops growing on Chilean soils is scarce. Soils in Chile have high contents of copper and manganese [2].
• The dataset includes crops and plants not commonly studied, such as 21 Chilean endemic tree species.

## 1 Objective

The Soil and Plant Nutrition Laboratory of Chilean Agricultural Research centre analyzes about 700 plant samples a year, serving farmers as a side job to their research work. The laboratory passes on the results directly to agronomist and consultants and archives the reports in digital format. It has been doing so since 2006. 'Previously, all reported were archived for two years in paper format then destroyed. Two years that equals the embargo period for the reported data.

Since 2006, lab reports have transitioned from occupying physical archive shelves to being stored indefinitely on a hard drive. As a result, they have no purpose after the embargo period. Recognizing this, we identified the digital storage unit as a means to organize and provide access to tissue analysis data from both wild plants and crops cultivated in the soils of the Chilean Central Valley. The dataset encompasses all records from 2006 to 2020, with the last records having reached the end of their embargo period.

## 2 Data Description

**Fig. 1:**
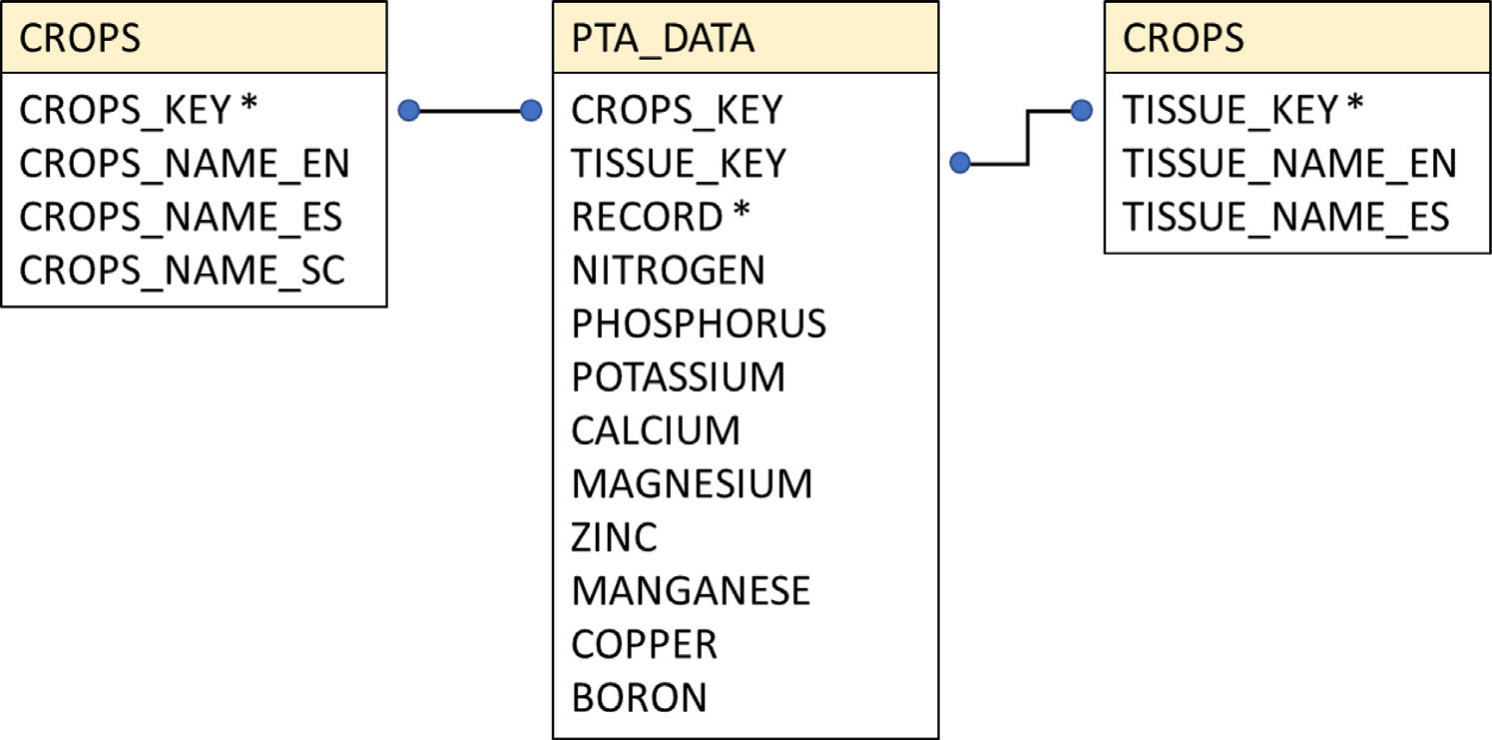
Dataset diagram. Asterisks indicate primary table keys. Black lines connecting blue points show entity relationships.

The dataset comprises one SQL file with three data tables (Fig. 1). The data tables are labeled CROPS, SAMPLED_TISSUE, and PTA_DATA. The CROPS data table has four columns. The first stands for a unique ID, which is also the CROPS table primary key. The other three columns have crops names in English (CROP_NAME_EN), Spanish (CROP_NAME_ES), and the scientific names (CROP_NAME_SC). Legitimate or *nomen conservandum* scientific names were included to facilitate translation to languages other than the two provided. The data table has 94 rows.

The SAMPLED_TISSUE data table has three columns. The first stands for a unique ID, which is also the table primary key. The other two columns have descriptors for the plant tissue on which the analyses were performed: one column has the tissue names in English (TISSUE_NAME_EN), the other in Spanish (TISSUE_NAME_ES). The data table has 14 rows.

The PTA_DATA data table has twelve columns and contain the plant tissue analysis data. The first two columns relate the PTA_DATA table with the other two by attaching to every row the crop ID and tissue ID that correspond to the analysis of that row. The third column assigns a unique ID to every entry of the PTA_DATA table. Of the remaining columns, five of them have six-digit float numbers with up to four decimal places (NITROGEN, PHOSPHORUS, POTASSIUM, CALCIUM, MAGNESIUM). The last four columns have signed integers (ZINC, MANGANESE, COPPER, AND BORON). The data table has 9175 rows.

Reported values for nitrogen, phosphorus, potassium, calcium and magnesium are expressed as%. Values for zinc, manganese, copper and boron in mg/kg.

## 3 Experimental Design, Materials and Methods

### 3.1 Laboratory reports

All laboratory reports available in the hard drive were systematized by date. They were all Microsoft Word document files named after the internal ID of the samples processed. There were 1540 single files from 2006 to 2020 in the hard drive. As the format of the reports changed during the years, sample data was exported manually to an Excel spreadsheet. Reports were discarded when: (a) crop and sampled tissue was unknown; or (b) the analytical method followed was not clear.

Crops and plant species names were harmonized, mainly to present them in lower case letters while keeping the first in upper case. As reported names were common names in Spanish, we retrieve manually scientific names from Tropicos.org [2] keeping only those that were legitimate or *conservandum*. The process revealed that some plants were reported under different common names, despite corresponding to the same species, variety or cultivar. It was the case for the Peruvian pepper (*Schinus molle* L.) and the Magellan barberry (*Berberis microphylla* K. Koch). One column was added to provide English common names.

Sampled tissues were harmonized, presenting them in lowercase letters and using singular nouns (e.g., replacing roots by root). A column was added to provide the English translation.

The Spreadsheet was split and exported to comma separated values (csv) to produce the three separated data tables comprised in the dataset. The csv files were imported to MariaDB and the dataset backed up using mysqldump. The backed-up file is the one distributed: it has a set of SQL statements that can be executed to reproduce the original database object definitions and table data.

### 3.2 Plant tissue analysis

All analysis reported were done following Kalra Reference Methods for Plant Analysis [3]. Nitrogen was determined by Kjeldahl method. Tissue samples were digested with sulfuric acid, salicylic acid and hydrogen peroxide. After the digestion, N—NH_4_ in the digestate is determined by NH_3_ distillation and titration. The remaining elements were measured after high-temperature oxidation of the organic matter and dissolution of the ash with hydrochloric acid. Concentrations of potassium, calcium, magnesium, zinc, manganese and copper in the digestates were determined by atomic absorption spectrometry. The same digestate was used to determine phosphorus concentrations by vanadium phosphomolybdate colorimetric reaction and boron by azomethine-H colorimetry.

### 3.3 Quality control

Since the year 2007 the Soil and Plant Nutrition Laboratory of INIA has participated in the Wageningen Evaluating Programs for Analytical Laboratories (WEPAL-QUASIMEME) proficiency tests. Along the years, the lab has scored within one standard deviation of WEPAL agreed values and use the samples as an internal laboratory quality assurance. Moreover, the WEPAL samples are used to calibrate internal samples used as control for routine analysis. In addition to controls, by protocol 20% of the samples are analyzed in duplicate, setting a value for the variation of a sample of less than 10% to accept the analyzes of a lot.

## Ethics Statements

The authors declare that all data included in the dataset has its embargo period expired and it has been anonymized to protect the farmers who requests the laboratory services.

The authors declare that no human or animal studies were conducted to collect the data included in this dataset.

## CRediT authorship contribution statement

**Fabio Corradini:** Conceptualization, Methodology, Validation, Resources, Data curation, Writing – original draft, Visualization, Project administration, Funding acquisition. **Francisco Casado:** Data curation. **Verónica Rojas:** Investigation, Validation, Data curation.

## Declaration of Competing Interest

The authors declare that they have no known competing financial interests or personal relationships that could have appeared to influence the work reported in this paper.

## Data Availability

INIA_PTA_2023 (Original data) (biblioteca.inia.cl).
